# Perseverance time of informal caregivers for people with dementia: construct validity, responsiveness and predictive validity

**DOI:** 10.1186/s13195-017-0251-0

**Published:** 2017-04-04

**Authors:** Anke Richters, René J. F. Melis, N. Job van Exel, Marcel G. M. Olde Rikkert, Marjolein A. van der Marck

**Affiliations:** 1 Department of Geriatric Medicine, Donders Institute for Brain Cognition and Behaviour, Radboud university medical center, PO Box 9101 (hp 925), Nijmegen, 6500 HB The Netherlands; 2 Radboudumc Alzheimer Center, Radboud university medical center, Nijmegen, The Netherlands; 3 Department of Geriatric Medicine, Radboud Institute for Health Sciences, Radboud university medical center, Nijmegen, The Netherlands; 4 Institute of Health Policy and Management, Erasmus University Rotterdam, Rotterdam, The Netherlands; 5 Erasmus School of Economics, Erasmus University Rotterdam, Rotterdam, The Netherlands

**Keywords:** Dementia, Informal care, Psychometric properties, Validity, Burden

## Abstract

**Background:**

Informal care is essential for many people with dementia (PwD), but it often results in a considerable burden for the caregiver. The perseverance time instrument integrates the aspect of perceived burden with the caregiver’s capacity to cope with the burden, in contrast to most available instruments, which measure solely the burden of caregiving. The aim of this study was to extend insight into psychometric properties of the perseverance time instrument, specifically the construct validity, responsiveness, and predictive validity, within the population of informal caregivers for PwD.

**Methods:**

Data from two studies among informal caregivers of community-dwelling PwD in the Netherlands were used. The first study included 198 caregivers from a single region in the Netherlands and lasted 1 year. The second was a cross-sectional nationwide study with 166 caregivers for PwD. Questionnaires of both studies included questions regarding demographics and informal care, perseverance time, and other informal caregiver outcomes (Caregiver Strain Index, Self-rated Burden scale, Care-related Quality of Life instrument, and visual analogue scale health scores). Construct validity and responsiveness were assessed using a hypothesis-testing approach. The predictive validity of demographic characteristics and perseverance time for living situation after 1 year (living at home, institutionalized, or deceased) was assessed with multivariable multinomial regression.

**Results:**

All but one of the hypotheses regarding construct validity were met. Three of five hypotheses regarding responsiveness were met. Perseverance time scores at baseline were associated with living situation after 1 year (*p* < 0.01), unlike age, sex, and relationship with PwD. Perseverance time strongly increased predictive power for living situation after 1 year (c-index between 0.671 and 0.775) in addition to demographic characteristics.

**Conclusions:**

This study supports previous findings regarding the construct validity of the perseverance time instrument and adds new evidence of good construct validity, responsiveness, and predictive validity. The predictive power of perseverance time scores for living situation exceeds the predictive power of other burden measures and indicates informal care as an important factor for maintaining the patient at home.

## Background

Informal care, which is nonprofessional care provided by people from a person’s social environment, is a large and crucial part of all necessary care for people with dementia (PwD), and it is usually provided by a partner or child [1]. As in most other developed countries, the vast majority of Dutch PwD live at home, evidencing the great demand for informal care. As the number of PwD is projected to rise substantially and demands on health care resources increase, it is becoming increasingly important to maintain adequate informal care to uphold quality care for community-dwelling PwD.

Hence, it is of utmost importance that those willing and able to provide informal care for a loved one with dementia can maintain the care situation for as long as possible. However, providing this care often comes with a considerable burden [2]. Their ability to maintain their caregiver role depends on their perceived burden as well as on their capacity to cope with this burden. This means that the burden of care has to be acceptable, given the physical, emotional, social, and financial capacities of the informal caregiver. Although the balance between burden and capacity to cope is crucial for maintaining informal care, the majority of informal care instruments assess solely the burden of care, from either an objective (hours spent on care) or a subjective (perceived burden) perspective [3].

The recently introduced perseverance time instrument integrates the aspects of perceived burden and ability to cope, and thus potentially provides valuable information to health care professionals and researchers about caregivers’ ability to maintain informal care. This instrument consists of one question that asks the informal caregiver to indicate the time she or he will be able to continue providing care under a hypothetically stable situation. Although earlier validation steps have been carried out and shown to have promising results (construct validity has been tested in a single population construct [4]), more information is desirable before widespread application [4–6]. For example, the instrument has not been thoroughly assessed for responsiveness and predictive validity. Therefore, the aim of this study was to extend construct validity assessment to a broader population and to investigate the responsiveness and predictive validity.

## Methods

### Data

Individual participant data of two questionnaire studies were used. The first dataset was derived from a longitudinal study (study A) of 198 informal caregivers of community-dwelling PwD, with paper questionnaires sent to their home addresses at baseline and after 1 year to be completed independently [4]. Informal caregivers were approached through a regional assessment agency with a registry of diagnosed PwD. This study was specifically set up to validate the perseverance time instrument. The second dataset was obtained from a cross-sectional study (study B) in which an online questionnaire, also to be completed independently, was sent to a sample representative of adults in the Netherlands in terms of age and sex [7]. A total of 1244 informal caregivers responded, 166 of whom reported providing informal care to a community-dwelling PwD and were selected for the present research. The subsequent selection of dementia informal caregivers is not necessarily representative of dementia informal caregivers in the Netherlands. We cannot analyze the selection mechanisms at play, because no information on the nonresponders is available, either for the whole group or for the subgroups of people who provided informal care for a PwD. However, no specific selection was applied by the researchers. Recruitment of informal caregivers for study A took place between September 2007 and March 2008, after which the caregivers were included in longitudinal data collection, and data collection of study B took place in October 2010.

### Measures

Demographic characteristics of caregivers and PwD they cared for included age, sex, relationship (child, partner, or other), and duration of informal care. In addition, the questionnaires included various measures of caregiver outcomes. Perseverance time measures the time for which a caregiver will be able to continue providing care if the caregiving situation remains as it currently is, and it includes six ordered answering categories: <1 week, 1 week–1 month, 1–6 months, 6 months–1 year, 1–2 years, and >2 years. The Care-related Quality of Life (CarerQol) instrument measures care-related quality of live and consists of the CarerQol-7D and the CarerQol-visual analogue scale (VAS) [8–10]. The CarerQol-7D comprises two positive and five negative dimensions of care-related burden to which caregivers can respond regarding their experience with the level score “no,” “some,” or “a lot.” A summary score reflecting care-related quality of life can be obtained by applying a tariff derived from the Dutch general population to each scoring option [11]. The CarerQol-VAS is a score tallied using a VAS representing caregiver’s general level of happiness (range 0–100, where higher scores reflect greater happiness). The Self-rated Burden scale (SRB) is an overall assessment of care burden with a single VAS (range 0–100, where higher scores indicate higher burden) [12]. The Caregiver Strain Index (CSI) assesses the caregiver’s negative caregiving experiences with 13 propositions to which the caregiver can indicate if the statements apply to their situation (yes/no; score range 0–13, where higher scores indicate higher burden) [13]. The objective burden was quantified as the average of hours per week spent on providing informal care, which is the sum of multiple informal care tasks (e.g., personal care, instrumental activities of daily living tasks, health care visits). Two additional VAS scores were included for the overall perceived health of the informal caregiver and of the PwD as rated by the caregiver (range from 0 for “worst imaginable health” to 10 for “best imaginable health”).

### Statistical analysis

For analysis of construct validity, data from study A (baseline data) and study B were analyzed both separately and conjointly using a meta-analytic approach. To assess construct validity of perseverance time (i.e., the degree to which scores are consistent with hypotheses) [14], we employed a hypothesis-testing approach with correlations between perseverance time and the other instruments. Hypotheses were constructed on the basis of the notion that perseverance time is an integral reflection of perceived burden of care and capacity to cope with this burden. CarerQol-7D, CSI, and SRB (all measuring subjective burden) were expected to be at least moderately to highly related with perseverance time. The measure of objective burden (less closely related) and CarerQol-VAS (a more general assessment of happiness) were both expected to be slightly more weakly associated. Health of the informal caregiver was thought to be related to the capacity to cope with burden and was thus expected to be associated. Health of the PwD was expected to be unrelated to the capacity to cope with the burden of the informal caregiver, because it is believed that this aspect relies mainly on intrinsic factors of the informal caregiver. Furthermore, health of the PwD is largely suboptimal in this patient group because it concerns elderly people who have dementia but often also comorbidities. It is expected that these health deficits may be partially associated with actual informal care tasks, which are only partially associated with perceived burden of the informal caregiver. Because this indirect association with only one aspect of the perseverance time construct, we expected negligible correlation between health of the PwD with perseverance time. Together, this resulted in the following hypothesized Spearman’s correlations, using the guidelines for strength described by Hopkins [15]: CarerQol-7D tariff (positive, moderate/strong correlation), CarerQol-VAS (positive, moderate), SRB (negative, moderate/strong), CSI (negative, moderate/strong), objective burden (negative, moderate), and VAS scores on health of informal caregiver (positive, weak/moderate) and health of PwD (no correlation). Random effects meta-correlations allowing for heterogeneity between both studies were calculated in the pooled dataset [16].

Responsiveness (also referred to as *longitudinal validity*)—that is, the degree to which changes in scores over time are consistent with hypotheses [17]—was assessed within study A. For informal caregivers who were included in both baseline and follow-up questionnaires (*n* = 74) [5], changes in scores over time were calculated. Hypotheses regarded correlations of 1-year change in perseverance time with CarerQol-VAS score, SRB, CSI, caregiver health VAS score, and objective informal care burden, and correlations were expected to be weak to moderate.

Predictive validity of baseline perseverance time regarding living situation of PwD (living at home, institutionalized, or deceased) after 1 year was assessed by means of three multinomial models. The lower three categories of perseverance time (<1 week, 1 week–1 month, and 1–6 months) were combined in these models because these were too few in number to separately provide sufficient power for the analysis (*n* = 0, *n* = 12, and *n* = 29, respectively). First, basic characteristics (age, sex, and relationship of informal caregiver and PwD) were selected as explanatory variables for living situation of PwD after 1 year. Second, a model was constructed with only perseverance time as an explanatory variable. Third, variables in these models were combined to assess the added value of perseverance time over basic characteristics. Last, SRB, CSI, and CarerQol-7D scores were added iteratively, instead of perseverance time, to the basic characteristics to compare the added predictive value of perseverance time relative to other burden measures. Predictive validity of these models was assessed through pairwise c-statistics between each couple of outcome categories and compared between models [18]. Analyses were conducted in SAS version 9.2 (SAS Institute, Cary, NC, USA) and R 3.1.1 (packages *meta* and *nnet*; R Foundation for Statistical Computing, Vienna, Austria) software.

## Results

### Study populations

The characteristics of the study populations are shown in Table 1. In both populations, the majority of informal caregivers were women. In study B, informal caregivers were more often children of PwD, whereas study A included mostly partners, hence the higher average age and higher proportion living with PwD.

**Table 1:** Characteristics of informal caregivers and people with dementia

	Study A (*n* = 198)	Study B (*n* = 166)
Caregiver		
Female sex (%)	67	55
Age, years, mean (SD)	66.6 (12.9)	49.5 (14.4)
Resides with people with dementia, %	59	17
Duration of informal caregiving, years		Median (IQR)
0	7%	3 (1–5)
1	27%	
2	20%	
3	18%	
4	14%	
5 or more	15%	
Informal care, h/week, median (IQR)	20 (8–50)	8 (5–18)
Relationship with people with dementia, %		
Spouse or partner	55	7
Child	37	54
Other	8	40
Perseverance time, %		
Less than 1 month	6	10
More than 1 month, less than 6 months	15	11
More than 6 months, less than 1 year	20	13
More than 1 year, less than 2 years	20	13
More than 2 years	40	54
CarerQol-7D tariff, mean (SD)	70.2 (19.2)	75.2 (20.4)
CarerQol-VAS, mean (SD)	6.4 (1.8)	7.0 (1.7)
CSI, median (IQR)	8 (6–10)	5 (2–7)
SRB, mean (SD)	6.0 (2.2)	4.8 (2.3)
VAS health score, mean (SD)	7.3 (1.6)	7.3 (1.7)
People with dementia		
Female sex, %	53	73
Age, years, mean (SD)	81.3 (6.6)	76.6 (17.0)
Health VAS score, mean (SD)	5.8 (1.9)	5.4 (2.0)

CarerQol-7D score ranges from 0 to 100, with higher scores indicating favorable situation; CarerQol-VAS score ranges from 0 to 10, with higher scores indicating favorable situation; CSI scores range from 0 to 18, with lower scores indicating favorable situation; SRB scores range from 0 to 10, with lower scores indicating favorable situation; and VAS health score ranges from 0 to 10, with higher scores indicating a more favorable situation. The category “Other” in relationship includes other family members, friends, neighbors, and acquaintances

*Abbreviations*: *CarerQol* Care-related Quality of Life instrument, *CSI* Caregiver Strain Index, *SRB* Self-rated Burden scale, *VAS* Visual analogue scale

### Construct validity and responsiveness

In study A, correlations were moderate to strong and statistically significant between perseverance time score and CarerQol-7D and CarerQol-VAS, SRB, and CSI (Table 2), and correlations were weak with objective burden and informal caregiver VAS health score. There was no significant correlation with PwD VAS health score. Data from study B showed similar results, except for correlation with informal caregiver VAS health score (not significant). Meta-correlations showed no relevant differences between both study populations, except for a significantly higher correlation with SRB in study A. All hypotheses were met, except for informal caregiver VAS health score, which was lower than expected.

**Table 2:** Correlation of perseverance time with related measures for assessment of construct validity and responsiveness

	Construct validity^a^			Responsiveness^b^
	Study A	Study B	Pooled data	Study A
CSI	−0.45^c^	−0.40^c^	−0.42^c^	0.27^d^
SRB	−0.62^c^	−0.36^c^	−0.50^c^	0.28^d^
CarerQol-7D	0.32^c^	0.46^c^	0.39^c^	N/A
CarerQol-VAS	0.23^c^	0.28^c^	0.25^c^	−0.23^d^
Objective burden	−0.24^c^	−0.28^c^	−0.26^c^	0.10
IC VAS health	0.19^c^	0.15	0.17^c^	−0.19
PwD VAS health	0.10	−0.01	0.05	N/A

*Abbreviations*: *CarerQol* Care-related Quality of Life instrument, *VAS* Visual analogue scale, *IC* Informal caregiver, *PwD* People with dementia, *SRB* Self-rated Burden scale, *CSI* Caregiver Strain Index, *N/A* Not applicable, because these measures were not included in the follow-up measurement

^a^ Correlations between actual scores

^b^ Correlations between change scores over time. Change in perseverance time was used as a positive or negative difference in number of ordered answering categories between follow-up and baseline. Change in other scores is used as continuous difference between follow-up and baseline

^c^ *p* < 0.05

^d^ *p* < 0.01

Change scores for perseverance time over 1 year of follow-up showed significant correlations only with the change scores on CarerQol-VAS, SRB, and CSI, and not with objective burden and VAS health scores of PwD and informal caregivers (Table 2). The observed correlations were weak to moderate.

### Predictive validity

Overall, 37% of PwD still lived at home 1 year after baseline measurement, 41% were institutionalized, and 21% were deceased. Proportions still living at home increased considerably over the increasing categories of perseverance time at baseline, from 8% for the answer category less than 1 month to 51% for the answer category more than 2 years (*p* < 0.001). The opposite was true for institutionalization, ranging from 67% in the lowest category to 30% in the highest.

The multivariable multinomial model without perseverance time showed that none of the basic characteristics were statistically associated with higher risk of either institutionalization or death, nor did any show strong effect estimates (Table 3). They jointly yielded very limited predictive value for living situation, as indicated by the pairwise c-indices (0.611–0.639). In contrast, perseverance time alone was significantly associated with higher risk of both institutionalization and death. When perseverance time was added to the model with basic characteristics, only perseverance time was statistically associated with higher risk of institutionalization and death and strongly increased predictive value (c-indices 0.671–0.775). When perseverance time was replaced with CSI, SRB, or CarerQol-7D, none of these measures yielded as great an increase in predictive value as perseverance time (highest c-indices obtained by CSI 0.62–0.73).

**Table 3:** Multivariable multinomial regression models to assess predictive validity of perseverance time (data from study A)

	Basic characteristics		Perseverance time		Basic characteristics and perseverance time	
	OR institutionalization	OR deceased	OR institutionalization	OR deceased	OR institutionalization	OR deceased
PwD sex						
Male	Reference	Reference	–	–	Reference	Reference
Female	0.76 (0.31–1.91)	1.29 (0.42-3.99)	–	–	0.79 (0.30–2.09)	1.28 (0.41–4.03)
Informal caregiver sex						
Male	Reference	Reference	–	–	Reference	Reference
Female	0.93 (0.38–2.29)	0.66 (0.20–2.09)	–	–	1.01 (0.39–2.65)	0.71 (0.22–2.33)
PwD age						
Per year	1.05 (0.98–1.12)	1.06 (0.97–1.15)	–	–	1.03 (0.96–1.10)	1.04 (0.96–1.13)
Informal caregiver age						
Per year	0.98 (0.93–1.04)	0.97 (0.91–1.04)	–	–	0.99 (0.94–1.05)	0.97 (0.92–1.05)
Relationship with PwD						
Partner	Reference	Reference	–	–	Reference	Reference
Child	1.19 (0.25–5.74)	0.66 (0.09-4.61)	–	–	2.39 (0.43–13.18)	1.06 (0.14–8.21)
Other	1.22 (0.27–5.53)	0.55 (0.06-4.88)	–	–	2.40 (0.45–12.02)	0.80 (0.09–7.49)
Perseverance time						
<6 months	–	–	Reference	Reference	Reference	Reference
6–12 months	–	–	0.37 (0.54–0.72)	0.20 (0.09–1.52)	0.16 (0.04–0.60)	0.32 (0.08–1.36)
1–2 years	–	–	0.13 (0.04–0.43)	0.12 (0.03–0.59)	0.08 (0.02–0.31)	0.12 (0.03–0.56)
>2 years	–	–	0.15 (0.03–0.29)	0.09 (0.04–0.55)	0.06 (0.02–0.21)	0.14 (0.04–0.54)
c-Index						
Home vs. institutionalization	0.639		0.689		0.775	
Home vs. deceased	0.611		0.659		0.717	
Institutionalization vs. deceased	0.631		0.571		0.672	

*PwD* People with dementia

## Discussion

The results of this study with multiple datasets and comprehensive assessment in a longitudinal setting support previous findings regarding the construct validity of the perseverance time instrument. The present study adds new evidence of good construct, responsiveness, and predictive validity. The results show adequate construct validity based on two separate study populations of informal caregivers for PwD as well as a pooled population of community-dwelling PwD. Moreover, we found moderate to good responsiveness. Analyses also showed considerably higher predictive value by perseverance time of living situation after 1 year than for basic characteristics such as age, sex, and relationship, as well as other burden measures, indicating high predictive validity.

Construct validity was assessed by testing hypotheses regarding correlations between perseverance time and other related constructs, based on the assumption that perseverance time incorporates subjective burden and capacity to cope with the burden. Earlier findings regarding the construct validity were promising [4]. This study adds to the evidence of high construct validity by supporting these previous findings in a new study. Furthermore, we were able to pool data from two previous studies, resulting in a more divergent population, with one of the study populations selected from among the general population. Although both populations concerned informal caregivers of community-dwelling PwD, characteristics showed that the care situation was slightly more burdensome in study A than in study B, possibly resulting from selection source (registry of formal help for dementia), and the relationship and residence with the PwD were also differently distributed. In general, the partner is the primary informal caregiver for the PwD and is usually of older age, like the PwD. The fact that study B used an online questionnaire to recruit informal caregivers may have resulted in selection of more informal caregivers who were younger, because these individuals are more likely to regularly use computers and take part in online questionnaires. This is indeed supported by the difference in informal caregiver characteristics between study A and study B, with study B consisting of, on average, younger informal caregivers, with a smaller proportion being the PwD’s partner. This provides more importance to the analysis performed regarding construct validity. Nevertheless, despite the divergence between both study populations, construct validity was equally well upheld in both studies, indicating a wide range of application opportunities. Study B was initiated in a sample representative of the adult population in the Netherlands in terms of age and sex. Owing to the selection of only informal caregivers for PwD among those who responded, the resulting study population might not be representative of all adult informal caregivers for PwD in the Netherlands. However, the major characteristics that may be relevant for this particular study population are well described in this study. Furthermore, unlike studies with prevalence estimates, for instance, the exact representativeness of the study population is less relevant for a validation study, because it is highly unlikely that the instrument will function differently in slightly different populations of informal caregivers for PwD.

The responsiveness of the perseverance time instrument had not been assessed before. Because application of this instrument is especially suitable in a research setting of a progressive disease, it is particularly useful to know whether the instrument accurately reflects changes over time, such as in the setting of use of the instrument or the longitudinal effects of determinants. Our results show that constructs that are theoretically the furthest from perseverance time indeed were not significantly correlated to change scores for perseverance time. This was in line with our expectations because there is already an expected deviation among scores as constructs only partially overlap. When looking at change scores, this deviation was expected to become even larger because different but related constructs do not necessarily change in the same direction and the same magnitude over time within a person. The fact that change in perseverance time significantly correlated with the subjective burden measures of CSI and SRB as well as the CarerQol-VAS indicates that perseverance time is sensitive to changes over time, supporting its use in longitudinal settings. It must be kept in mind that statistical power to show significant correlations in this analysis was impaired by the fact that there were only 74 observations, and the majority of informal caregivers reported no change in perseverance time, resulting in little dispersion on which to base correlation.

In an earlier study using the same data, researchers looked at the percentages of informal caregivers who anticipated the perseverance time correctly by considering whether the patient still lived at home after 1 year [5]. This gave some first indications of predictive validity, which was further complemented in the present study by employing more sophisticated methods. First, the alternatives to the situation of living at home (i.e., being institutionalized or deceased) were separated by employing multinomial models because perseverance time may be differently associated with each of these alternatives. Second, we did not dichotomize indicated perseverance time as being more or less than 1 year, but instead kept separate answer categories in the analyses. Last, we additionally provided insight in the predictive value of perseverance time for predicting the three separate outcomes by calculating pairwise c-statistics.

Our results show that a longer indicated perseverance time was associated with higher risk of both institutionalization and death of PwD after 1 year. This indicates that perseverance time predicted institutionalization and death. Interestingly, it showed that this single-question instrument had high accuracy in predicting PwD who still lived at home after 1 year and those who were institutionalized or deceased. This was even the case in addition to known characteristics (age and sex of PwD and informal caregiver and their relationship), unlike other perceived burden measures. This indicates that the perseverance time instrument indeed measures a construct that transcends perceived burden. We have now assessed the predictive value of perseverance time scores for the events of institutionalization and death to underscore the predictive validity of the instrument as such. Additional studies are required to assess the added value of other potentially useful predictors for these events, such as severity of dementia. Which other predictors are relevant is largely dependent on the setting of the research or, as in the case of the present study, which information is readily available.

On the basis of the present and previous findings, it is clear that the perseverance time instrument validly reflects the construct to be measured (i.e., an integration of burden of informal care with the capacity to cope with the burden). This short and easy-to-use instrument therefore constitutes a strong and valuable tool in care and research on informal caregiving for PwD. However, because validity is dependent on aspects such as setting and population, and not a characteristic belonging to the instrument itself, further validation in other caregiving settings and populations is recommended.

## Conclusions

The results of this study with multiple datasets and comprehensive assessment in a longitudinal setting support previous findings regarding the construct validity of the perseverance time instrument. This study adds new evidence of good construct validity, responsiveness, and predictive validity. The predictive power of perseverance time scores for living situation exceeds the predictive power of other burden measures and indicates informal care as an important factor for maintaining the patient at home.

## Abbreviations

CarerQol: Care-related Quality of Life instrument
CSI: Caregiver Strain Index
IC: Informal caregiver
PwD: People with dementia
SRB: Self-rated Burden scale
VAS: Visual analogue scale
